# Supplementary material for: Safinamide Improves Non-Motor Symptoms Burden in Parkinson’s Disease: An Open-Label Prospective Study
Source: Brain Sci. 2021 Mar 2;11(3):316. doi: 10.3390/brainsci11030316 (PMC7999475; doi:10.3390/brainsci11030316)
Supplement: Supplementary file 1 [file brainsci-11-00316-s001.pdf]

Table 1. SM. Change in the score of the NMSS and its domains between the visits of the study: V1 (N=50), V2 (N=47), V3 (N=45), V4 (N=44). .

	**V1**	**V2**	**V3**	**V4**	**p[a]**	**p[b]**	**p[c]**	**p[d]**
**NMSS total score**	97.48 ± 43.70	65.98 ± 40.10	59.40 ± 32.34	59.91 ± 35.49	**<0.0001**	**<0.0001**	**<0.0001**	0.525
- Cardiovascular	9.58 ± 2.46	6.29 ± 10.64	5.09 ± 8.23	6.72 ± 11.94	0.268	0.056	0.118	0.500
- Sleep / fatigue	36.08 ± 21.77	23.58 ± 20.97	18.33 ± 14.36	23.15 ± 18.12	**0.002**	**<0.0001**	**<0.0001**	0.912
- Mood / apathy	34.42 ± 29.89	19.47 ± 21.51	16.94 ± 17.29	14.49 ± 19.63	**<0.0001**	**<0.0001**	**<0.0001**	0.604
- Perceptual symptoms	4.33 ± 8.67	3.02 ±7.04	2.66 ± 4-32	2.84 ± 5.88	0.630	0.896	0.515	0.640
- Attention / memory	17.50 ± 17.09	14.83 ± 18.86	14.26 ± 16.93	13.32 ± 18.19	**0.026**	0.070	0.150	0.959
- Gastrointestinal symptoms	19.61 ± 18.01	13.94 ± 16.94	14.07 ± 14.59	13.13 ± 13.39	**0.010**	**0.013**	**0.013**	0.764
- Urinary symptos	42.72 ± 30.41	30.32 ± 24.96	28.21 ± 27.99	30.62 ± 23.94	**0.003**	**0.002**	**0.001**	0.701
- Sexual dysfunction	28.25 ± 35.69	24.29 ± 36.91	27.87 ± 35.62	25.28 ± 33.58	0.784	0.867	0.249	0.474
- Miscellaneous	33.33 ± 20.73	22.78 ± 16.29	19.12 ± 15.01	18.99 ± 14.03	**<0.0001**	0.084	**0.001**	0.235
Dose of safinamide (mg/day)	N. A.	53.84 ± 13.49	96.15 ± 13.49	98.72 ± 8.00				

P values were computed using the Wilcoxon signed-rank test. The results represent mean ± SD. Domains of the NMSS were expressed as a percentage to be able to establish comparisons on their severity between them; p[a], V4 vs V1; p[b], V3 vs V1; p[c], V2 vs V1; p[d], V4 vs V2. N. A., Not applicable.

NMSS, Non-Motor Symptoms Scale.
